# Comparative Gene Expression Analysis in WM164 Melanoma Cells Revealed That *β*-*β*-Dimethylacrylshikonin Leads to ROS Generation, Loss of Mitochondrial Membrane Potential, and Autophagy Induction

**DOI:** 10.3390/molecules23112823

**Published:** 2018-10-30

**Authors:** Nadine Kretschmer, Alexander Deutsch, Christin Durchschein, Beate Rinner, Alexander Stallinger, Juan Carlos Higareda-Almaraz, Marcel Scheideler, Birgit Lohberger, Rudolf Bauer

**Affiliations:** 1 Institute of Pharmaceutical Sciences, Department of Pharmacognosy, University of Graz, Universitaetsplatz 4/1, 8010 Graz, Austria; nadine.kretschmer@uni-graz.at (N.K.); christin.durchschein@uni-graz.at (C.D.); 2 Department of Hematology, Internal Medicine, Medical University Graz, Auenbruggerplatz 15, 8036 Graz, Austria; alexander.deutsch@medunigraz.at; 3 Department for Biomedical Research, Medical University Graz, Roseggerweg 48, 8036 Graz, Austria; beate.rinner@medunigraz.at (B.R.); alexander.stallinger@medunigraz.at (A.S.); 4 Institute for Diabetes and Cancer (IDC), Helmholtz Zentrum München, German Research Center for Environmental Health, 85764 Neuherberg, Germany; juan.higareda@helmholtz-muenchen.de (J.C.H.-A.); marcel.scheideler@helmholtz-muenchen.de (M.S.); 5 Joint Heidelberg-IDC Translational Diabetes Program, Heidelberg University Hospital, 69120 Heidelberg, Germany; 6 German Center for Diabetes Research (DZD), 85764 Neuherberg, Germany; 7 Department of Orthopedics and Trauma, Medical University of Graz, Auenbruggerplatz 5, 8036 Graz, Austria; birgit.lohberger@medunigraz.at

**Keywords:** *β*-*β*-dimethylacrylshikonin, melanoma, p62, autophagy, ROS generation, mitochondrial membrane potential

## Abstract

Skin cancer is currently diagnosed as one in every three cancers. Melanoma, the most aggressive form of skin cancer, is responsible for 79% of skin cancer deaths and the incidence is rising faster than in any other solid tumor type. Previously, we have demonstrated that dimethylacrylshikonin (DMAS), isolated from the roots of *Onosma paniculata* (Boraginaceae), exhibited the lowest IC_50_ values against different tumor types out of several isolated shikonin derivatives. DMAS was especially cytotoxic towards melanoma cells and led to apoptosis and cell cycle arrest. In this study, we performed a comprehensive gene expression study to investigate the mechanism of action in more detail. Gene expression signature was compared to vehicle-treated WM164 control cells after 24 h of DMAS treatment; where 1192 distinct mRNAs could be identified as expressed in all replicates and 89 were at least 2-fold differentially expressed. DMAS favored catabolic processes and led in particular to p62 increase which is involved in cell growth, survival, and autophagy. More in-depth experiments revealed that DMAS led to autophagy, ROS generation, and loss of mitochondrial membrane potential in different melanoma cells. It has been reported that the induction of an autophagic cell death represents a highly effective approach in melanoma therapy.

## 1. Introduction

According to the WHO [1], the incidence of skin cancers has been increasing over the past decades. Globally, 2–3 million new cases of non-melanoma (basal cell carcinoma and squamous cell carcinoma) and 132,000 malignant melanoma skin cancers occur each year, which corresponds to one third of all diagnosed cancers. Basal cell carcinoma is slowly growing and unlikely to spread to distant areas. Squamous carcinoma is able to metastasize [2]. Melanoma, the most aggressive form of skin cancer originates from melanocytes and accounts for only 4% of all skin cancers. However, it is responsible for 79% of all skin cancer deaths. The incidence of malignant melanoma is rising faster than in any other solid tumor type [3], and overall, it is responsible for about 2% of all cancer deaths in the United States [4]. Moreover, it is estimated that a 10% decrease in ozone levels will result in 4500 additional melanoma cases. At a very early stage (Tis), the survival rate is almost 100%. Even in superficial melanomas below 1 mm tumor depth (T1), the prognosis remains at over 90% of survival [5]. However, at an advanced stage, the vertical growth phase, or depth of more than 1 mm, survival rates drop dramatically which leaves melanoma as one of the most aggressive and incurable types of solid cancer. One of the central pathways dysregulated in most melanoma is the RAS-RAF-MEK-ERK-MAP kinase pathway. In 66 %, a BRAF mutation can be found, typically caused by a single substitution (V600E). Mutated BRAF proteins have an increased kinase activity leading to uncontrolled cell growth, and therefore, cancer development [6]. WM164 melanoma cells were originally isolated from a melanoma metastasis in the right upper arm of a 22-year-old male with stage IV superficial spreading melanoma. This cell line exhibits this typical V600E mutation, and therefore, was chosen for this study [7].

Natural products always played a crucial role in the discovery of new therapeutics. Today, about 80% of all small-molecule anti-cancer drugs are natural products per se, based thereon or mimicking natural products [8]. In our search for new promising candidates, *β*-*β*-dimethylacrylshikonin (DMAS) isolated from the roots of *Onosma paniculata* Bureau and Franchet. (Boraginaceae) arose as such a molecule. Traditionally, these roots are used for the treatment of measles and other eruptive exanthema, skin infections, eczema, burns, cancer, scalds, and constipation [9]. Related species with the same class of bioactive compounds (shikonin and/or alkannin derivatives) are officially listed in the Chinese, Japanese, and Korean Pharmacopoeia, and for five tumor types in the Tibet-China pharmacopoeia [10,11]. Shikonin derivatives have been shown to possess a broad pharmacological spectrum, including wound-healing, anti-inflammatory, and anti-cancer activity [12,13,14]. In previous studies, we have shown that DMAS was our main and most active isolated compound. It was able to reduce the viability of cancer cells, especially melanoma cell lines, and induced apoptosis and cell cycle arrest [15,16]. In this study, we used a microarray-based approach to investigate which genes were up- or down-regulated under DMAS treatment in WM164 cells. The most interesting effects were examined in more detail and compared to two other melanoma cell lines.

## 2. Results and Discussion

### 2.1. Comparative Gene Expression Analysis Revealed 31 Distinct mRNAs as at Least 2-Fold Significantly Differentially Expressed with Sequestosome 1 (p62) mRNA as Largest Change

**Figure 1:**
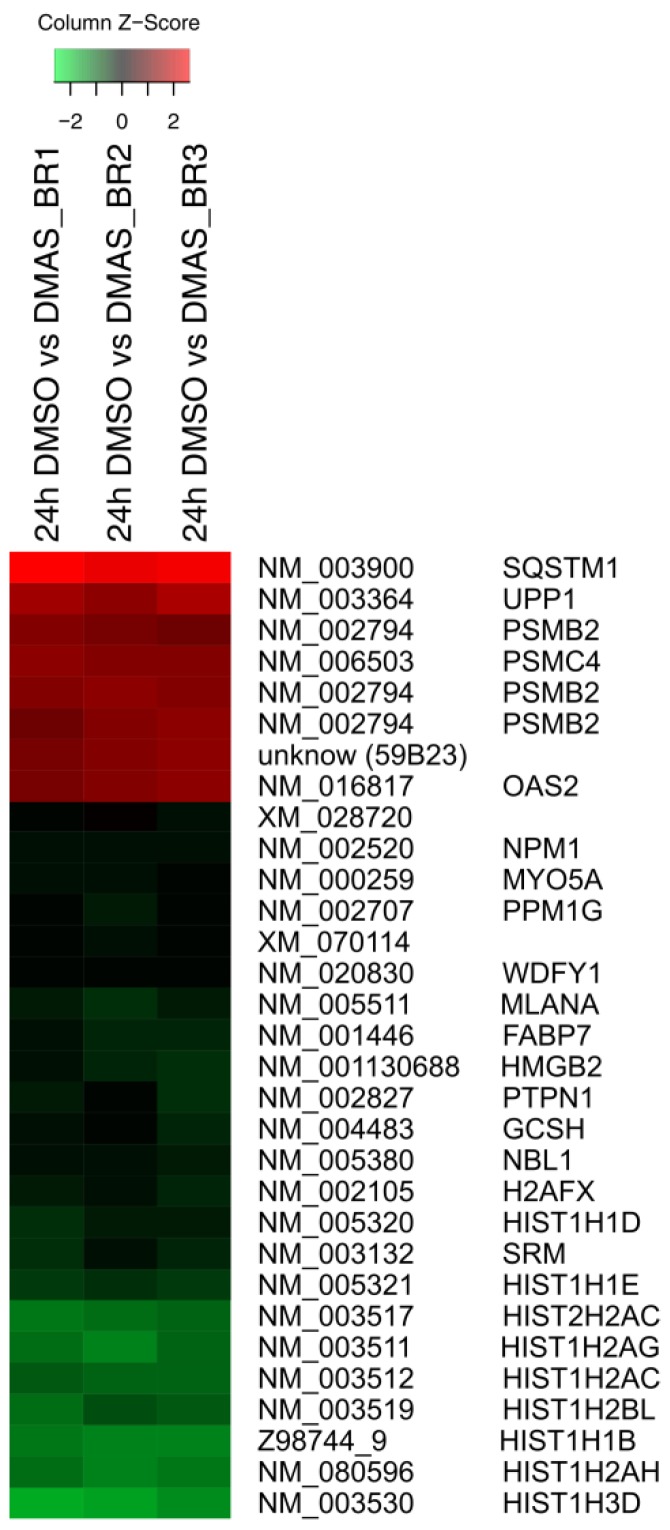
At least twofold significantly differentially expressed gene expression levels (log_2_ ratios) in WM164 cells after 24 h treatment with dimethylacrylshikonin (DMAS) colored according to the legend at the top and sorted to the degree of up- and down-regulation. In all three biological replicates, 31 distinct mRNAs were identified as significantly differentially expressed as calculated by the one-sample *t*-test and Benjamini-Hochberg correction.

**Figure 2:**
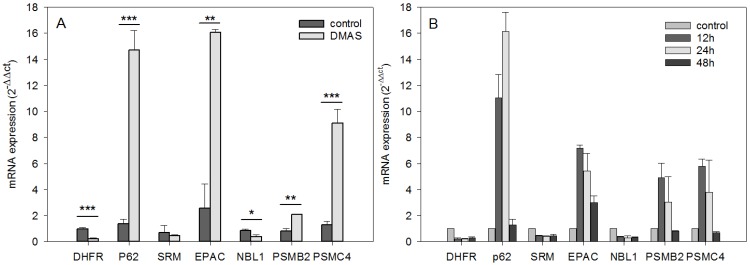
RT-qPCR validation and kinetics of genes identified by the transcriptome profiling (mean ± sem, *n* = 3). (**A**): total RNA of WM164 cells was again used to validate the expression levels of six differentially expressed genes. Except for *SRM*, the results were again statistically significant and the mRNA expression was changed in the same direction (up- vs. downregulation) confirming the microarray results (* *p* < 0.5, ** *p* < 0.1, *** *p* < 0.01). (**B**): For the same genes, kinetics of the gene expression was obtained by quantifying the mRNA amount after 12 h, 24 h, and 48 h of DMAS treatment. The strongest changes were found after 12 h and 24 h.

Using microarray and in-depth bioinformatics analyses, we comprehensively investigated the gene expression upon a 24 h treatment of DMAS (8.3 µM, which was the prior determined IC_50_ value [16]) in three biological replicates and compared to vehicle-treated (0.5% DMSO) WM164 cells. In total, 3021 distinct mRNAs were identified as expressed in at least 2 of 3 biological replicates, and 1192 distinct mRNAs as expressed in all three replicates. Out of these, 317 distinct mRNAs were identified as 1.5-fold differentially expressed in all three biological replicates, and 135 distinct mRNAs as 1.8-fold differentially expressed (data not shown). However, we focused on genes which were at least 2-fold differentially expressed. In all three biological replicates, 89 distinct mRNAs were identified and tested for significance using the one-sample t-test followed by the Benjamini-Hochberg correction for multiple testing. This resulted in 31 distinct mRNAs (Figure 1). The strongest upregulation was found for *sequestosome 1* (*sqstm1*, *p62*, NM_003900) (24.42 fold upregulated); the strongest downregulation for the *h3 histone family member b* (*h3fb*, NM_003530) (18.78 fold downregulated). *P62* is rich in protein-interacting sequences and plays an important role in cell growth, survival, and mitosis. It has also been shown to be a regulator and substrate of autophagy, and, as a consequence, is a central regulator of tumorigenesis [17]. The results were validated by real-time semi quantitative PCR (RT-qPCR). The same RNA was used for validation and seven genes were chosen. Except for *spermidine synthase* (*SRM*), the changes were again statistically significant. The strongest upregulations were again found for *p62*, and moreover, *Rap1 Guanine-Nucleotide-Exchange Factor Directly Activated by CAMP* (*EPAC*) and *proteasome* (*prosome*, *macropain*) *subunit*, *beta type*, *2* (*psmb2*); the strongest downregulation was for *dihydrofolate reductase* (*DHFR*) and *neuroblastoma*, *suppression of tumorigenicity* (*NBL1*) (Figure 2A). In addition, kinetics for these genes was performed. RNA levels were quantified after 12 h, 24 h, and 48 h. The strongest changes were found after 12 h and 24 h (Figure 2B).

### 2.2. DMAS Favors Catabolic Processes

**Figure 3:**
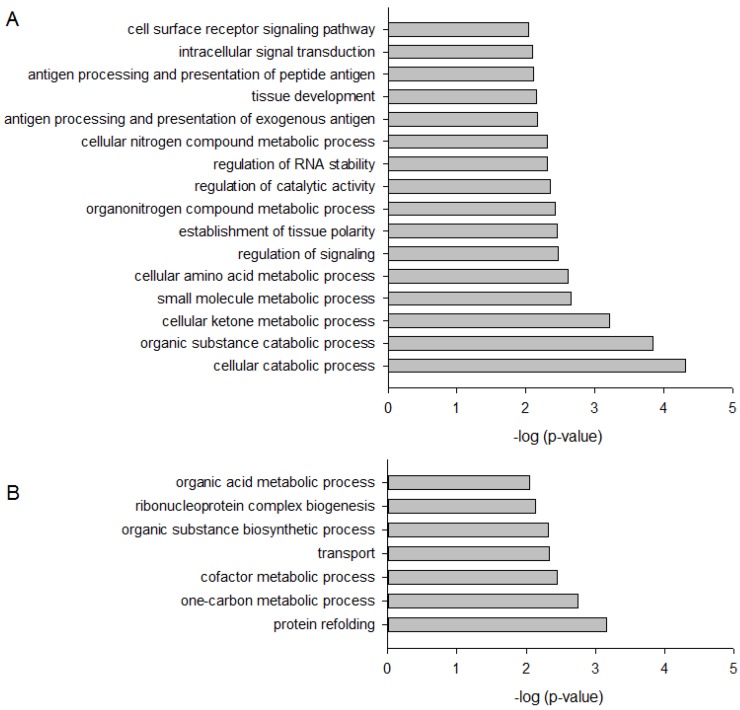
Bar plot depicting significantly up- (**A**) and down-regulated (**B**) Gene Ontology (GO) Biological Process in WM164 cells when treated with 8.3 µM DMAS for 24 h.

To identify cellular processes and pathways influenced by DMAS, GO term analysis using the Biological Process domain in level 3 (Figure 3) and pathway analysis (Table 1 and Table 2) were performed using ConsensusPathDB [18]. As shown in Figure 3, 16 biological processes were upregulated by DMAS and seven were downregulated. The strongest upregulation was found for “cellular catabolic process” (GO:0044248) and “organic substance catabolic process” (GO:1901575), both belonging to the biological process “catabolic process” (GO:0009056). This suggests that the cells enhance breakdown of (organic) substances. “Cellular catabolic process” also comprises the process of autophagy in which p62 plays an important role. In addition, the strongest downregulated process was “protein refolding” (GO:0042026), indicating that unfolded or misfolded proteins were no longer restored. Twenty-two pathways were found to be upregulated (Table 1), and twenty-four were downregulated (Table 2). The strongest upregulation was found for the Nrf2 pathway, which can be explained as a consequence of p62 activation. Activation of p62 leads to sequestration of Keap1 into autophagosomes, which in turn, leads to Nrf2 activation, which is the key signaling molecule within this pathway [19]. In general, the Keap1-Nrf2-ARE pathway plays an important role in defense mechanisms against many types of stress and is involved in the development of inflammatory diseases and cancer [19]. In healthy cells, Nrf2 activation promotes chemopreventive effects. In fully malignant cancer cells, enhanced Nrf2 activity, caused for example by BRAF mutations as found in WM164 cells, increases cancer chemoresistance and tumor growth [20,21], and therefore, is oncogenic. This reflects the complex role of this pathway. As a consequence, the question arose if pharmacological compounds which activate this pathway can lead to cancer growth and/or resistance. To date, this question is not clearly answered. Synthetic triterpenoids are already in use for patients with advanced malignancies, well-tolerated, and also known to activate the Nrf2 signaling pathway. For example, it has been shown that RTA 405 did not reduce the sensitivity of cancer cells towards doxorubicin and cisplatin, despite increased Nrf2 activity. Moreover, such compounds even enhanced the cytotoxicity of chemotherapeutics [22]. Similar results were also obtained for bardoxolone methyl in combination with carboplatin and paclitaxel in a mouse lung cancer model [23]. Therefore, there are indications that such a mechanism could even provide an advantage when combined with standard therapeutics to protect normal cells from drug-associated side effects [22,23].

### 2.3. DMAS Increased the Expression of the Autophagy-Associated Protein LC3B-II

**Figure 4:**
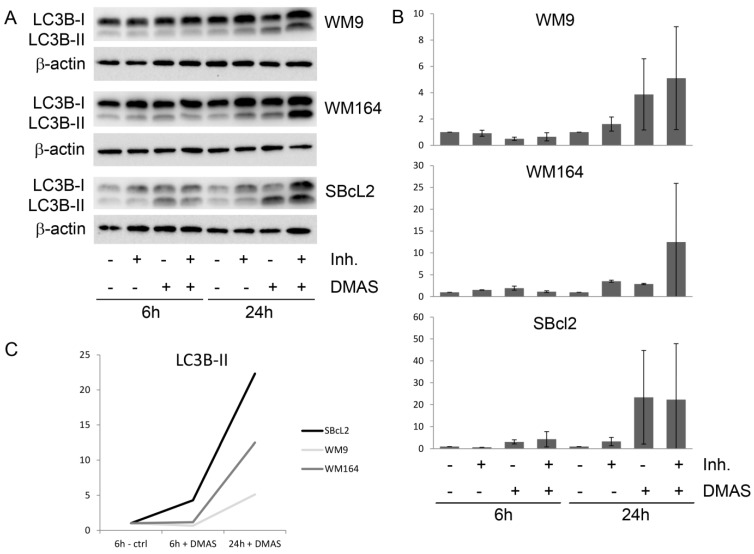
DMAS induced autophagy in human melanoma cells after 6 h and 24 h. (**A**): Western blot analysis for the expression of LC3BI-II. The lysosomal protease inhibitors E64d and pepstatin A (Inh.) blocked the autophagic flux and inhibited the degradation of LC3B-II. One representative blot out of three is shown. (**B**): The densiometric quantification of the relative LC3B-II protein expression (mean ± sem, *n* = 3). (**C**): Time course of the relative expression of LC3B-II.

**Figure 5:**
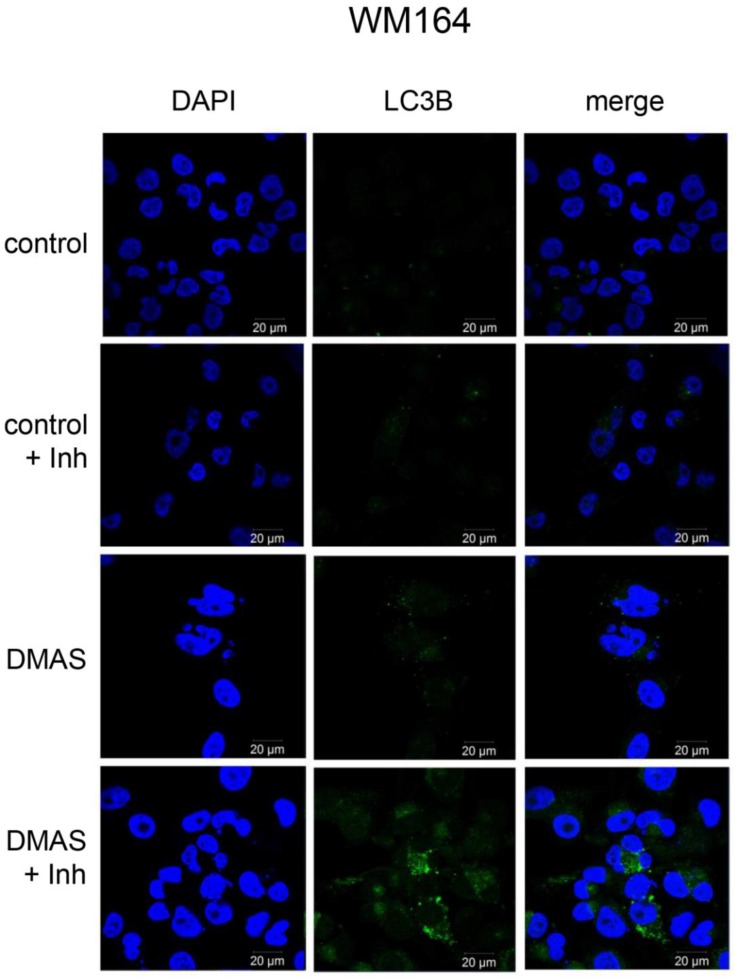
Effects of DMAS treatment on LC3B immunostaining in WM164 melanoma cells. Double immunolabeling with DAPI (blue) and anti-LC3B (green) antibodies was performed as described in the methods section. The lysosomal protease inhibitors E64d and pepstatin A (Inh) blocked the autophagic flux and inhibited the degradation of LC3B-II (bar: 20 µm). Vehicle-treated cells were measured as controls.

Since the largest change in mRNA expression was found for p62, which is a regulator and substrate of autophagy, we monitored the expression of the autophagy-associated protein LC3BI-II in three different melanoma cells (WM164, WM9, and SBcl2). Each cell line was treated with the IC_50_ of DMAS, as determined in previous experiments [16]: WM164: 8.3 µM, WM9: 2.7 µM, and SBcl2: 1.1 µM. We decided to choose these concentrations because they covered a broad concentration range, which made it difficult to treat all cell lines with the same concentration. SBcl2 cells originate from an early state of cancer progression (cutaneous melanoma, radial growth phase) and carry a mutation in NRAS [24]. WM9 cells represent another cell line derived from metastases located at the left axillary node and are also BRAF mutated [25]. When autophagy occurs, the microtubule-associated protein LC3B localizes to isolation membranes leading to the formation of autophagosome membranes. LC3B-I, the cellular form, converts to LC3B-II and the amount of LC3B-II becomes a marker for the formation of autophagosomes. Degradation of LC3B-II can partially be inhibited by adding lysosomal protease inhibitors, such as E64d and pepstatin A, whereas, LC3B-I is not affected [26]. Therefore, we performed the experiments additionally in the presence of these inhibitors. Western blot experiments revealed a significant upregulation of LC3B-II in all three cell lines (Figure 4). The densiometric quantification of the relative LC3B protein expression is shown in Figure 4B. Figure 4C shows the time course of the quantitative expression of LC3B-II. As mentioned earlier, BRAF mutations can lead to enhanced Nrf2 activity, and as a consequence, enhanced autophagic flux [20,21,27]. When comparing all control cells (WM164, WM9, and SBcl2) in Figure 4A, it can be seen that WM9 and WM164 (both BRAF mutated) show higher levels of basal autophagy than SBcl2 cells. Increased autophagy of these cell lines was also reported previously [28]. This, in turn, is an explanation of why the relative increase of autophagy is stronger in SBcl2 cells than in the other cell lines, with already enhanced basal autophagy (Figure 4C). The change in the subcellular distribution of LC3B can also be visualized by immunofluorescence microscopy. The characteristic pattern of LC3B puncta can be observed in autophagic cells stained with anti-LC3B antibody, whereas, untreated control cells revealed only a small amount of LC3B positive cells (Figure 5). The effect of lysosomal protease inhibitors E64d and pepstatin A can also be observed. Inhibition of autophagy prevented LC3B-II degradation, which, subsequently, resulted in LC3B-II accumulation (Figure 5, last row). Our results indicated that DMAS leads not only to apoptosis and cell cycle arrest, but also induces autophagy. Autophagic cell death has been reported to be highly effective in the therapy of melanoma [29]. Moreover, it has been shown that activation of apoptosis and autophagy can result in a synergistic cytotoxicity in melanoma cells [30].

### 2.4. DMAS Leads to ROS Generation and Loss of Mitochondrial Membrane Potential

**Figure 6:**
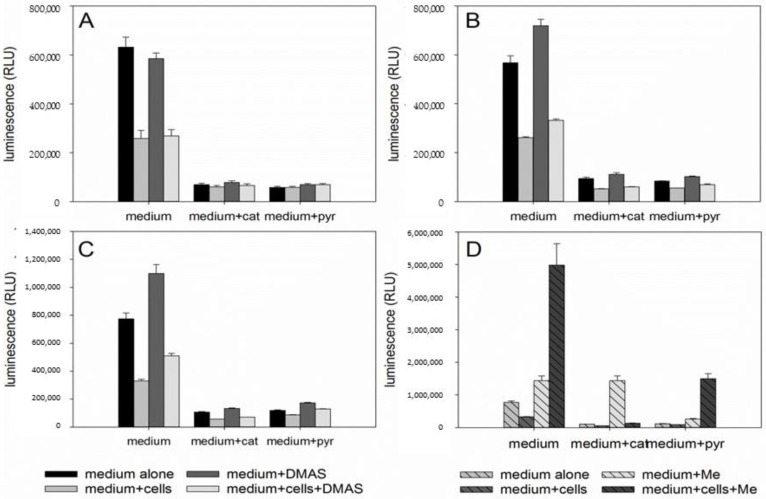
Results of the ROS-Glo™ assay. **A**: SBcl2, **B**: WM9, and **C**: WM164 cells treated with the respective IC_50_ of DMAS in the absence or presence of 1 µg/mL catalase (cat) or 1 mM pyruvate (pyr) for 4 h. **D**: WM164 cells treated with menadione (positive control) in the absence or presence of 1 µg/mL catalase (cat) or 1 mM pyruvate (pyr). Results are displayed as mean ± sem (*n* = 6).

**Figure 7:**
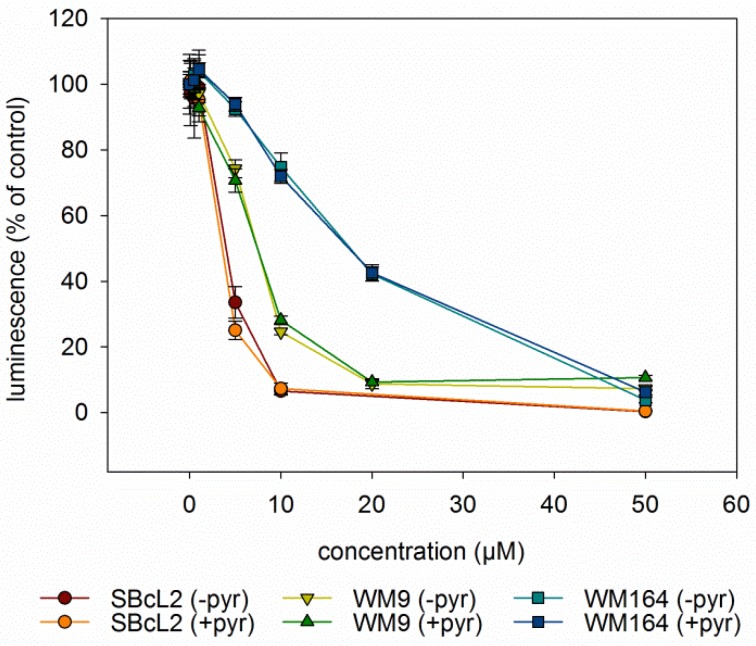
Results of the CellTiterGlo^®^ Assay (mean ± sem, *n* = 6). All three cell lines were treated with various concentrations of DMAS for 72 h in the absence (-pyr) or presence (+pyr) of 1 mM pyruvate. Thereby, the addition of pyruvate did not change the effects of DMAS.

**Figure 8:**
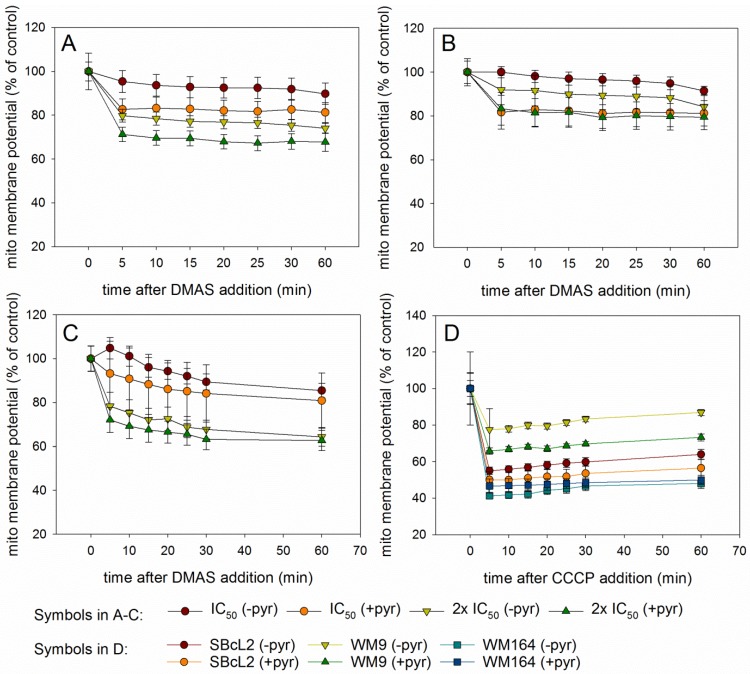
Results of the Mito-ID^®^ assay (mean ± sem, *n* = 6). (**A**): SBcl2, (**B**): WM9, and (**C**): WM164 cells treated with the respective IC_50_ or 2 × IC_50_ of DMAS for up to 60 min in the presence (+pyr) or absence (-pyr) of 1 mM pyruvate. The mitochondrial membrane potential decreased concentration dependently in presence and absence of pyruvate. (**D**): Effects of the positive control CCCP (10 µM) on mitochondrial membrane potential of SBcl2, WM9, and WM164 cells.

Since autophagy is often regulated by the levels of reactive oxygen levels (ROS) [31,32], we investigated whether DMAS led to generation of ROS. Generation of oxidative stress is an important mechanism of cytotoxic drugs. However, in *in vitro* test systems it has to be remembered that components of the cell culture medium can affect the results. For example, it is known that polyphenols can produce H_2_O_2_ in cell culture media independent of cells [33,34]. Moreover, cells have mechanisms to eliminate ROS which can lead to lower ROS levels in “medium plus cells” wells, compared to “medium only” wells. Sodium pyruvate neutralizes H_2_O_2_, and therefore, can reveal if cytotoxicity based on ROS generation is mediated by internal or external ROS production. In addition, catalase converts H_2_O_2_ to O_2_ and H_2_O and can also be added to eliminate external ROS [35]. Therefore, we measured the amount of ROS in different scenarios: “medium alone”, “medium plus cells”, “medium plus DMAS”, and “medium plus cells plus DMAS”. Moreover, we used three different preparations for the component “medium”: RPMI-1640 medium without pyruvate or catalase, RPMI-1640 medium supplemented with 1 mM pyruvate, and RPMI-1640 medium supplemented with 100 µg/mL (350 U/mL) catalase (in each case, RPMI-1640 medium was additionally supplemented with 2% FBS and 1% Pen/Strep). Cells were treated with their respective IC_50_ of DMAS for 4 h. As positive control, we used menadione (Vitamin K3), which is known to induce ROS cell-dependence [35,36]. The following effects were observed in all three cell lines (Figure 6): Adding cells to the medium reduced the amount of H_2_O_2_. When DMAS was added to the medium, the amount of H_2_O_2_ increased indicating that DMAS also led to external ROS generation. In both cases, these effects were also observed in the presence of pyruvate and catalase, but the total ROS amount was less due to the radical scavenging activities of pyruvate and catalase. When combining medium, cells, and DMAS, the amount of H_2_O_2_ was increased compared to “medium+cells” indicating that DMAS led also to the generation of intracellular ROS [35]. The same effects were also found for menadione. To exclude the possibility that DMAS exerts its cytotoxicity by extracellular H_2_O_2_ production, we performed the CellTiter-Glo^®^ Assay in the presence and absence of 1 mM pyruvate and compared the effects of DMAS. This assay quantifies the amount of ATP, which is an indicator of metabolically active cells. As shown in Figure 7, the effects of DMAS did not change in the presence of pyruvate indicating that generation of extracellular ROS, as observed in Figure 6A–C, did not significantly contribute to the cytotoxicity of DMAS (IC_50_ values in absence and presence of 1 mM pyruvate: SBcl2: 4.1 ± 0.3 µM and 3.2 ± 0.1 µM; WM9: 6.6 ± 0.3 µM and 6.6 ± 0.2 µM; WM164: 19.2 ± 3.1 µM and 18.1 ± 2.4 µM, respectively). These results were in agreement with a previous study showing that shikonin and DMAS increased ROS levels in colon cancer cells, and as a consequence, sensitized them to ionizing radiation [37]. Finally, we wanted to know if the generation of ROS leads to a loss of the mitochondrial membrane potential, since the mitochondria are a major cellular source of ROS [32,38], and if the presence of pyruvate influences that effect. In the case of shikonin, it has been demonstrated that it directly targets mitochondria, generates ROS, and therefore, leads to cell cycle arrest and apoptosis in different types of cancer [39,40,41,42]. Thereby, shikonin was also able to enhance chemotherapeutic sensitivity of the cells [42]. Cell cycle arrest and apoptosis were also observed in our previous study [16]. We treated the cells with the respective IC_50_ or double IC_50_ for 60 min, and measured the mitochondrial membrane potential every 5 mins for the first 30 min, and in addition, after 60 min in the presence and absence of pyruvate. As shown in Figure 8A–C, a moderate time- and concentration- dependent loss of the mitochondrial membrane potential could be observed. The effect was not diminished by the addition of pyruvate. CCCP (carbonyl cyanide 3-chlorophenylhydrazone), a known mitochondrial membrane potential perturbation agent, was used as positive control (Figure 8D). Our findings indicate that ROS generation and loss of the mitochondrial membrane potential were basic reasons for cell cycle arrest, apoptosis, as well as autophagy induction. 

### 2.5. Cytotoxicity of DMAS Against Non-Tumorigenic Cells Depends on Cell Type

Despite promising results, it has to be remembered that DMAS was also cytotoxic towards non -tumorigenic cell lines in our previous study [16]. Therefore, we also determined the IC_50_ values of DMAS against another well-established non-tumorigenic cell line (HEK-293), and two non-tumorigenic primary cell lines (juvenile and adult fibroblasts). We used the XTT viability assay as reported previously [16] to be able to directly compare the values. As shown in Table 3, the cytotoxicity of DMAS varies depending on cell type. The divergent cellular responses can be explained by the different origin of the cells. For example, in the case of fibroblasts, it has been shown that fibroblasts of different origin exhibit different transcriptional patterns and behavior [43]. Nevertheless, cytotoxicity towards healthy cells is a general problem in cancer therapy and leads to undesirable adverse reactions in patients. One example is doxorubicin, a commonly used chemotherapeutic. It exhibits the same or even higher cytotoxicity against HEK-293 cells than against several tumor cells [44,45]. To overcome this problem, smart loaded nanoparticles could be a solution. Blood vessels of tumors are leaky, and lymphatic drainage within tumors is poor allowing nanoparticles to accumulate in the tumor and the drug to be released [46]. In the case of shikonin, it has already been reported for gliomas that shikonin-loaded nanoparticles improved the anti-tumor effects *in vitro*. Moreover, these particles accumulated in the brain of rats [47]. In addition, self-assembled nanomicelles of clotrimazole improved drug delivery and apoptosis, and inhibited tumor progression in melanoma [48]. However, the development, characterization and detailed testing of such nanoparticles goes beyond the scope of the current work.

## 3. Material and Methods

### 3.1. Isolation and Identification of DMAS

Roots of *Onosma paniculata* Bureau and Franchet (Boraginaceae) were acquired at the medicinal plant market in Kunming, China, and identified by genomic analysis of the ITS2 region of nuclear DNA, and trnL-F region of plastid DNA as reported previously [15]. DMAS was isolated and identified as previously reported in Reference [16]. In brief, freshly ground roots were extracted with petroleum ether by exhaustive Soxhlet extraction. DMAS was isolated by using preparative HPLC consisting of a Varian R PrepStar SD-1 (Agilent Technologies, Santa Clara, CA, USA) with Dynamax R solvent delivery system and an absorbance detector model UV-1, and by using a VDSpher 100 RP18 column (250 × 25 mm, 10 μm). The mobile phase was water (A) and acetonitrile (B) using the following gradient: 0−45 min: 70−100% B; 45−60 min: 100% B. DMAS was identified by NMR and CD measurements, as in Reference [16]. Out of 400 mg extract, 48.6 mg DMAS was isolated. Its purity was analyzed by HPLC and NMR and was >95 %.

### 3.2. Cell Culture 

WM164, WM9, and SBcl2 cells were cultured in RPMI 1640 medium (Gibco, Invitrogen, Vienna, Austria) supplemented with 2% fetal bovine serum (FBS, Gibco) and 1% penicillin-streptomycin solution (Pen/Strep, Gibco^®^). HEK-293 cells were cultured in Dulbecco’s Modified Eagle Medium: Nutrient Mixture F-12 (DMEM/F12, Gibco^®^), 2 mM L-glutamine, 10% FBS, and 1% Pen/Strep. Human juvenile and adult fibroblasts were kindly provided by Ass. Prof. Dr. Beate Rinner (Medical University of Graz), and cultured in Dulbecco’s Modified Eagle Medium (DMEM), 2mM L-glutamine and 10% FBS. All cells were kept at 37 °C in a humidified 5% CO_2_ atmosphere and passaged at 90% confluence.

### 3.3. Sample Preparation for Microarray-Based Transcription Profiling

Three independent cell culture experiments were performed to obtain three biological replicates. Cultured WM164 cells were harvested by trypsinization and diluted to 400,000 cells/mL. From this cell suspension, 40 mL were seeded into a 175 cm² cell culture flask and grown overnight to allow the cell to adhere. Cells were then treated with 8.3 µM DMAS for 24 h, harvested by trypsinization, and washed twice with PBS (Gibco). Finally, the cell pellet was shock frozen in liquid nitrogen and stored at −80 °C before RNA was extracted. Vehicle-treated (0.5% DMSO) control cells served as reference cells.

### 3.4. RNA Extraction and RT-qPCR

Total RNA was extracted using Trizol according to the manufacturer’s protocol (Invitrogen, Carlsbad, CA, USA). cDNA was synthesized using 1 µg total RNA and the RevertAid™ H Minus First Strand cDNA Synthesis Kit (Fermentas, Darmstadt, Germany). Real-time semi quantitative PCR (RT-qPCR) for GAPDH (QT00079247), PPIA (QT01006285), HPRT1 (QT00059066), DHFR (QT016687930), p62 (QT00095676), SRM (QT00013258), EPAS (QT00069587), NBL1 (QT01004794), PSMB2 (QT02505769), and PSMC4 (QT01006859) using commercially available primer assays (Qiagen, Hilden, Germany—ids are provided in brackets of each respective gene) was performed using an ABI Prism 7000 Detection system (Applied Biosystems, Foster City, CA, USA). PCR reactions were performed in triplicates of 20 µL each. Reaction mix contained 1× QuantiNova® SYBR Green PCR Kit (Qiagen), 1mM forward and reverse primer (Eurofins Genomic, Ebersberg, Germany) or 1x QuantiTect Primer Assay (Qiagen), 4 μL cDNA (diluted 1:20) and aqua bidest. up to 20 µL, respectively. GAPDH, PPIA, and HPRT, possessing a high correlation coefficient (Spearman rho > 0.85 and *p* < 0.05), served as housekeeping genes. Results were expressed as relative units based on the calculation of 2^−ΔΔCT^, giving the relative amount of target gene normalized to the endogenous control (geometric mean of the three housekeeping genes) and relative to a low cytoplasmic NR4A1 expressing DLBCL specimen. The cycling protocol included 34 cycles with 2 min activation at 95 °C, followed by a denaturation step for 5 s at 95 °C, and an annealing/extension step for 10 s at 60 °C. Melt curve analysis served to identify the different reaction products including nonspecific ones.

### 3.5. Microarray-Based Transcription Profiling

The human oligonucleotide probe set (MWG) with 29,550 50-mer DNA oligonucleotides enabled the detection of 15,539 distinct RefSeq mRNAs, as well as non-RefSeq-annotated transcripts (provided by the manufacturer). Lyophilized oligonucleotides (in 384-well plates) were spotted on epoxy-coated glass slides (Nexterion, Jena, Germany) as previously described in Reference [49]. To analyze the effects of DMAS on the transcriptome, 15 µg of total RNA of 3 biological replicates of WM164 cells treated with DMAS or DMSO (as control) were reverse transcribed to generate cDNAs with incorporated aminoallyl-Uridine. Labeling was performed with fluorescent, amino-reactive Cy5 and Cy3 dyes for 1 h. Labeled cDNA from DMAS-treated cells was combined with the respective labeled cDNA from DMSO-treated cells in hybridization buffer (50% formamide, 5 × SSC, 0.1% SDS), denatured (95 °C/3 min) and applied to the slide for hybridization (39 °C, >16 h). After washing and drying, slides were scanned with a GenePix 4000B microarray scanner (Axon Instruments Inc., Union City, CA, USA) at 10 μm resolution using GenePix Pro 4.1 software (Axon Instruments Inc.). The resulting TIFF images were used to generate raw data results, which were pre-processed to filter out low intensity, saturated, and inhomogenous spots. Furthermore, the raw data was background corrected, normalized by global mean and dye-swap pairs using ArrayNorm as in Reference [50], and then exported as text-file containing log_2_ transformed ratios of gene expression. Data visualization, as well as filtering for present values and differential expression, was conducted with Genesis software, as outlined in Reference [51]. The day 0 and day 9 datasets contained 5340 and 4515 features that were (i) associated with a RefSeq transcript and (ii) detected in 3 or 4 biological replicates, respectively. Only these transcripts were considered for further analysis. Then, 3021 distinct mRNAs were identified as transcribed in at least 2 of 3 biological replicates, with 1192 distinct mRNAs expressed in all 3 biological replicates.

### 3.6. Microarray Data Analysis

Pathway analysis was performed using significant differentially expressed genes. Enrichment was done employing ConsensusPathDB, as in Reference [18] looking in gene ontology GO [52] and pathways, as defined by all the databases. Parameters were set for humans using the HGNC symbol, with a minimum overlap of three genes and a *p*-value threshold of 0.01. 

### 3.7. Western Blot

For autophagy investigations, 2 mL of a 150,000 cells/mL cell suspension (SBcl2, WM9, and WM164 cells) were seeded in each well of a 6-well plate and grown for 24 h to allow the cells to adhere. Afterwards, cells were treated with their respective previously determined IC_50_ for 6 h or 24 h: SBcl2: 1.1 µM, WM9: 2.7 µM, and WM164: 8.3 µM, as described in Reference [16]. Vehicle-treated (0.5% DMSO) cells served as control. Simultaneously, each sample was in parallel treated with the autophagy inhibitors pepstatin A (10 µg/ml, Sigma Aldrich, St. Louis, MO, USA) and E64d (10 µg/mL, Sigma Aldrich), which blocked autophagic flux and inhibited the degradation of LC3B-II [26]. Whole cell protein extracts were prepared by treatment with lysis buffer (50 mM Tris-HCl pH 7.4, 150 mM NaCl, 50 mM NaF, 1 mM EDTA, 10% NP-40, 1% Triton-X, and protease inhibitors), subjected to SDS-PAGE (10 or 12%) and blotted onto the PVDF membrane (Roth, Karlsruhe, Germany). Primary antibodies against LC3B I-II and β-actin were purchased from Cell Signaling Technology (Cell Signaling Technology, Danvers, MA, USA). Blots were developed using a horseradish peroxidase-conjugated secondary antibody (Dako, Jena, Germany) at room temperature for 1 h and the Amersham™ ECL™ prime Western blotting detection reagent (GE Healthcare, Chicago, IL, USA), in accordance with the manufacturer‘s protocol. Chemiluminescence signals were detected with the ChemiDocTouch Imaging System (BioRad Laboratories Inc., Herkules, CA, USA) and images were processed with the ImageLab 5.2 Software (BioRad Laboratories Inc.). Each Western blot experiment was performed in three biological replicates using three different cell passages.

### 3.8. Immunofluorescence

Regarding fluorescence microscopy, 500 µL of a 100,000 cells/mL cell suspension (SBcl2, WM9, and WM164) were seeded into each well of culture slides (FALCON® 4 chamber polystyrene vessel tissue culture treated glass slides; Corning Incorporated, Corning, NY, USA) and after 24 h of incubation, treated for another 24 h with the respective IC_50_ of DMAS: SBcl2: 1.1 µM, WM9: 2.7 µM, and WM164: 8.3 µM in the presence and absence of the lysosomal protease inhibitors E64d and pepstatin A (10 µg/ml; both Sigma Aldrich) [26]. Afterwards, cells were washed with PBS, fixed with 4% formaldehyde in PBS for 15 min at room temperature and again washed with PBS. Subsequently, cells were permeabilized for 10 min at 4 °C with ice-cold methanol. Cells were blocked 5 min at room temperature with UltraVision Protein Block (Thermo Fisher Scientific, Waltham, MA, USA) and treated with the primary antibody against LC3B purchased from Cell Signaling Technology or rabbit-IgG (Linaris Biologische Produkte, Dossenheim, Germany), for negative controls overnight at 4 °C. After an incubation for 2 h at room temperature with Alexa Fluor^®^ 488-conjugated AffiniPure Goat Anti-Rabbit IgG secondary antibody (Jackson Immunoresearch, Suffolk, UK), nuclei were counterstained and slides were mounted with Vectashield Mounting Medium with DAPI (Vector Laboratories, Burlingame, CA, USA). Cells were stored in the dark and viewed at a 510 LSM Meta confocal microscope (Zeiss, Vienna, Austria) using excitation at 405 nm and detection with a BP 420–480 nm filter for the nuclear stain. Binding of the LC3B antibody was measured by excitation at a wavelength of 488 nm, and detection with a LP 505 nm filter. ZEN 2009 Software was used to capture and process images.

### 3.9. ROS-Glo™ H_2_O_2_ Assay

The ROS-Glo™ H_2_O_2_ Assay from Promega (Mannheim, Germany) was performed in accordance with the manufacturer’s instructions. Specifically, 10,000 cells/well were seeded in white 96 well plates and grown for 24 h to allow the cells to adhere. Afterwards, medium was replaced by 75 µL RPMI medium, RPMI medium with 1 mM sodium pyruvate, or RPMI medium with 100 µg/mL (350 U/mL) catalase (Sigma-Aldrich) each containing 2% FBS and 1% Pen/Strep. Subsequently, 5 µL of test compound stock solution (DMAS or menadione as positive control) and 20 µL H_2_O_2_ Substrate solution was added and incubated for 4 h. In addition, the amount of ROS was also measured in medium alone and medium plus test compound (without cells). Finally, 100 µL of ROS-Glo™ Detection Solution was added to each well, incubated for 20 min, and luminescence was recorded on a Hidex Sense plate reader (Hidex, Turku, Finland).

### 3.10. CellTiter-Glo^®^ Luminescent Cell Viability Assay

The CellTiter-Glo^®^ Luminescent Cell Viability Assay was purchased from Promega and performed in accordance with the manufacturer’s instructions. In brief, 100 µL of 100,000 cells/mL were seeded in white 96 well plates and grown for 24 h to allow the cells to adhere. On the next day, medium was replaced by RPMI medium or RPMI medium containing 1mM pyruvate, and DMAS was added in different concentrations (0.5 to 50 µM) for 72 h. Thereafter, the plate was equilibrated to room temperature for 30 min and 100 µl of CellTiter-Glo^®^ Reagent was added. The plate was mixed on an orbital shaker for 2 min and incubated at room temperature for another 10 min to stabilize the luminescent signal. Luminescence was then recorded on a Hidex Sense plate reader.

### 3.11. MITO-ID^®^ Membrane Potential Detection Kit

The MITO-ID^®^ Membrane potential detection kit was purchased from Enzo Life Sciences, Inc. (Lausen, Switzerland) and performed as recommended by the manufacturer. In brief, 20,000 cells in 100 µL medium were seeded in black 96 well plates and incubated for 24 h to allow the cells to adhere. The next day, medium was replaced by fresh RPMI medium with or without 1 mM pyruvate, and 100 µL of MITO-ID^®^ MP Dye Loading Solution was added for 30 min before DMAS or CCCP (positive control) was added as well. In both cases, fluorescence was measured using a Hidex Sense plate reader (Ex = 4 85 nm, Em = 590 nm). 

### 3.12. XTT Assay

IC_50_ values of DMAS against non-tumorigenic cells were determined using the cell proliferation kit II (XTT) (Roche Diagnostics, Mannheim, Germany; cat. no. 11 465 015 001) as reported previously in References [15,16], and in accordance with the manufacturer’s instructions. Absorbance was recorded using a Hidex Sense Microplate Reader (Hidex, Turku, Finland).

### 3.13. Statistical Analysis

Microarray: In order to calculate changes in gene expression between vehicle-treated control and DMAS treated cells, differentially expressed genes were tested with a one-sample *t*-test (null hypothesis: log2[R/G] = 0), followed by a Benjamini-Hochberg correction for multiple testing to control the False Discovery Rate (FDR). Real-time RT-qPCR: Significant differences between the samples were calculated using the student’s *t*-test in case of normal distribution, and the Mann Whitney U test in case of non-normally distributes expression values. IC_50_ values: Values were calculated using SigmaPlot 13.0 (Systat Software Inc., Chicago, IL, USA), the four-parameter logistic curve, and at least two different cell passages each tested in three independent wells. Results were expressed as mean ± sem. *p*-Values < 0.05 were considered as statistically significant.

## 4. Conclusions

DMAS was previously found as the most active component of the roots of *Onosma paniculata*. In this study, we investigated the effects of DMAS on melanoma cells in more detail using a global transcriptome based approach. As a result, we found that DMAS favored catabolic processes indicating that the cells increase breakdown of (organic) substances. *Sesquestome1* (*p62*) was identified as the gene with the strongest elevation in expression which is a central player in the process of autophagy. Therefore, we investigated in more detail if DMAS induces autophagy. DMAS increased the amount of the autophagy-associated protein LC3B-II, which is an indicator for autophagy induction. Furthermore, as ROS levels are often regulators of autophagy and the mitochondria the main intracellular source of ROS, we hypothesized that DMAS leads to ROS generation and the loss of mitochondrial membrane potential. Indeed, DMAS led to both: ROS generation and a decline in the mitochondrial membrane potential. Thereby, the effects were not significantly changed by adding extracellular ROS scavengers, such as sodium pyruvate, indicating that ROS is mainly generated within the cells. Nevertheless, DMAS also exhibited cytotoxicity towards different non-tumorigenic cells. The effects varied depending on cell type. To overcome this problem, smart-loaded nanoparticles could represent a future solution.

In summary, our results demonstrated that DMAS leads not only to apoptosis and cell cycle arrest as shown in our previous studies [16], but also enhances autophagy. Therefore, a basic reason for the observed effects was the generation of ROS species, followed by a breakdown of the mitochondrial membrane potential. The autophagy mediated mode of cell death has previously been reported to be highly effective in melanoma treatment.

## Figures and Tables

**Table 1:** Pathway over-representation analysis of significantly upregulated genes.

Pathway	Set Size	Candidates	*p*-Value	Q-Value
NRF2 pathway	142	3 (2.1%)	0.0004	0.0057
Proteasome-Homo sapiens (human)	44	2 (4.5%)	0.0009	0.0057
Apoptosis-related network due to altered Notch3 in ovarian cancer	53	2 (3.8%)	0.0013	0.0057
TLR JNK	63	2 (3.2%)	0.0018	0.0057
IL-1 JNK	63	2 (3.2%)	0.0018	0.0057
TLR p38	64	2 (3.1%)	0.0019	0.0057
Proteasome Degradation	64	2 (3.1%)	0.0019	0.0057
DroToll-like	65	2 (3.1%)	0.0019	0.0057
IL-1 NFkB	65	2 (3.1%)	0.0019	0.0057
IL-1 p38	67	2 (3.0%)	0.0021	0.0057
TNF	67	2 (3.0%)	0.0021	0.0057
TLR NFkB	70	2 (2.9%)	0.0022	0.0057
Hedgehog	72	2 (2.8%)	0.0024	0.0057
Notch	79	2 (2.6%)	0.0028	0.0062
Nuclear Receptors Meta-Pathway	316	3 (0.9%)	0.0037	0.0076
CD4 T cell receptor signaling-NFkB cascade	95	2 (2.1%)	0.0041	0.0080
UCH proteinases	107	2 (1.9%)	0.0051	0.0093
TGF-beta super family signaling pathway canonical	115	2 (1.7%)	0.0060	0.0099
Wnt Canonical	120	2 (1.7%)	0.0064	0.0099
Wnt Mammals	120	2 (1.7%)	0.0064	0.0099
CD4 T cell receptor signaling	128	2 (1.6%)	0.0074	0.0109
B cell receptor signaling	134	2 (1.5%)	0.0080	0.0113

**Table 2:** Pathway over-representation analysis of significantly downregulated genes.

Pathway	Set Size	Candidates	*p*-Value	Q-Value
Metabolism of amino acids and derivatives	328	5 (1.5%)	0.0008	0.0086
Nucleotide Metabolism	19	2 (10.5%)	0.0008	0.0086
S-Adenosylhomocysteine (SAH) Hydrolase Deficiency	20	2 (10.0%)	0.0009	0.0086
Methionine Metabolism	20	2 (10.0%)	0.0009	0.0086
Methionine Adenosyltransferase Deficiency	20	2 (10.0%)	0.0009	0.0086
Glycine N-methyltransferase Deficiency	20	2 (10.0%)	0.0009	0.0086
Hypermethioninemia	20	2 (10.0%)	0.0009	0.0086
Methylenetetrahydrofolate Reductase Deficiency (MTHFRD)	20	2 (10.0%)	0.0009	0.0086
Homocystinuria-megaloblastic anemia due to defect in cobalamin metabolism, cblG complementation type	20	2 (10.0%)	0.0009	0.0086
Cystathionine Beta-Synthase Deficiency	20	2 (10.0%)	0.0009	0.0086
Methionine De Novo and Salvage Pathway	21	2 (9.5%)	0.0010	0.0087
One Carbon Metabolism	28	2 (7.1%)	0.0019	0.0142
Trans-sulfuration and one carbon metabolism	31	2 (6.5%)	0.0022	0.0158
Thiopurine Pathway, Pharmacokinetics/Pharmacodynamics	32	2 (6.2%)	0.0024	0.0158
TGF-beta receptor signaling activates SMADs	34	2 (5.9%)	0.0027	0.0167
Metalloprotease DUBs	37	2 (5.4%)	0.0032	0.0176
Selenoamino acid metabolism	132	3 (2.3%)	0.0032	0.0176
Metabolism	2035	11 (0.5%)	0.0035	0.0181
Cysteine and methionine metabolism-Homo sapiens (human)	45	2 (4.4%)	0.0047	0.0229
The citric acid (TCA) cycle and respiratory electron transport	171	3 (1.8%)	0.0067	0.0302
Pathogenic Escherichia coli infection-Homo sapiens (human)	55	2 (3.6%)	0.0070	0.0302
Pathogenic Escherichia coli infection	56	2 (3.6%)	0.0072	0.0302
Role of Calcineurin-dependent NFAT signaling in lymphocytes	58	2 (3.4%)	0.0077	0.0310
Folate Metabolism	66	2 (3.0%)	0.0099	0.0381

**Table 3:** IC_50_ values of DMAS against several non-tumorigenic cell lines (72 h of treatment, mean ± sem, *n* = 6). MRC-5: human lung fibroblasts; HEK-293: human embryonic epithelial cells; jFib: primary juvenile fibroblasts; aFib: primary adult fibroblasts. * Results previously reported in Reference [16].

Cell Line	MRC-5	HEK-293	jFib	aFib
IC_50_ value (µM)	2.4 ± 0.4 *	9.4 ± 1.4	1.8 ± 0.8	6.9 ± 1.1

## References

[1] Skin Cancers. http://www.who.int/uv/faq/skincancer/en/index1.html.

[2] Singh S., Zafar A., Khan S., Naseem I. (2017). Towards therapeutic advances in melanoma management: An overview. Life Sci..

[3] Aamdal S. (2011). Current approaches to adjuvant therapy of melanoma. Eur. J. Cancer.

[4] Middleton M.R., Lee S.M., Arance A., Wood M., Thatcher N., Margison G.P. (2000). O6-methylguanine formation, repair protein depletion and clinical outcome with a 4 h schedule of temozolomide in the treatment of advanced melanoma: Results of a phase II study. Int. J. Cancer.

[5] Bichakjian C.K., Halpern A.C., Johnson T.M., Foote Hood A., Grichnik J.M., Swetter S.M., Tsao H., Barbosa V.H., Chuang T.Y., Duvic M. (2011). Guidelines of care for the management of primary cutaneous melanoma. American Society of Dermatology. J. Am. Acad. Dermatol..

[6] Davies H., Bignell G.R., Cox C., Stephens P., Edkins S., Clegg S., Teague J., Woffendin H., Garnett M.J., Bottomley W. (2002). Mutations of the BRAF gene in human cancer. Nature.

[7] Smalley K.S., Haass N.K., Brafford P.A., Lioni M., Flaherty K.T., Herlyn M. (2006). Multiple signaling pathways must be targeted to overcome drug resistance in cell lines derived from melanoma metastases. Mol. Cancer Ther..

[8] Newman D.J., Cragg G.M. (2016). Natural products as sources of new drugs from 1981 to 2014. J. Nat. Prod..

[9] Zhongzhen Z. (2004). An illustrated Chinese Materia Medica in Hong Kong.

[10] The State Pharmacopoeia Commission of the People’s Republic of China (2000). Pharmacopoeia of the People´s Republic of China.

[11] Duke J.A., Ayensu S. (1985). Medicinal plants of China.

[12] Papageorgiou V.P., Assimopoulou A.N., Couladouros E.A., Hepworth D., Nicolaou K.C. (1999). The chemistry and biology of alkannin, shikonin and related naphthazarin natural products. Angew. Chem. Int. Ed..

[13] Chen X., Yang L., Oppenheim J.J., Howard M.Z. (2002). Cellular pharmacology studies of shikonin derivatives. Phytother. Res..

[14] Andújar I., Rios J.L., Giner R.M., Recio M.C. (2013). Pharmacological properties of shikonin—A review of literature since 2002. Planta Med..

[15] Rinner B., Kretschmer N., Knausz H., Mayer A., Boechzelt H., Hao X.J., Heubl G., Efferth T., Schaider H., Bauer R.J. (2010). A petrol ether extract of the roots of *Onosma paniculatum* induces cell death in a caspase dependent manner. J. Ethnopharmacol..

[16] Kretschmer N., Rinner B., Deutsch A.J., Lohberger B., Knausz H., Kunert O., Blunder M., Boechzelt H., Schaider H., Bauer R. (2012). Naphthoquinones from *Onosma paniculata* induce cell-cycle arrest and apoptosis in melanoma Cells. J. Nat. Prod..

[17] Moscat J., Diaz-Meco M.T. (2012). P62: A versatile multitasker takes on cancer. Trends Biochem. Sci..

[18] Kamburov A., Stelzl U., Lehrach H., Herwig R. (2013). The ConsensusPathDB interaction database: 2013 update. Nucl. Acids Res..

[19] Jiang T., Harder B., Rojo de la Vega M., Wong P.K., Chapman E., Zhang D.D. (2015). P62 links autophagy and Nrf2 signaling. Free Radic. Biol. Med..

[20] Kansanen E., Kuosmanen S.M., Leinonen H., Levonen A.L. (2013). The Keap1-Nrf2 pathway: Mechanisms of activation and dysregulation in cancer. Redox. Biol..

[21] Sporn M.B., Liby K.T. (2012). NRF2 and cancer: The good, the bad and the importance of context. Nat. Rev. Cancer.

[22] Probst B.L., McCauley L., Trevino I., Wigley W.C., Ferguson D.A. (2015). Cancer cell growth is differentially affected by constitutive activation of NRF2 by KEAP1 deletion and pharmacological activation of NRF2 by the synthetic triterpenoid, RTA 405. PLoS ONE.

[23] Liby K.T. (2013). Synthetic triterpenoids can protect against toxicity without reducing the efficacy of treatment with carboplatin and paclitaxel in experimental lung cancer. Dose Response.

[24] Hwang B.J., Adhikary G., Eckert R.L., Lu A.L. (2018). Chk1 inhibition as a novel therapeutic strategy in melanoma. Oncotarget.

[25] Satyamoorthy K., Li G., Gerrero M.R., Brose M.S., Volpe P., Weber B.L., van Belle P., Elder D.E., Herlyn M. (2003). Constitutive Mitogen-activated Protein Kinase Activation in Melanoma Is Mediated by Both BRAF Mutations and Autocrine Growth Factor Stimulation. Cancer Res..

[26] Mizushima N., Yoshimori T. (2007). How to interpret LC3 immunoblotting. Autophagy.

[27] Corazzari M., Rapino F., Ciccosanti F., Giglio P., Antonioli M., Conti B., Fimia G.M., Lovat P.E., Piacentini M. (2015). Oncogenic BRAF induces chronic ER stress condition resulting in increased basal autophagy and apoptotic resistance of cutaneous melanoma. Cell Death Diff..

[28] Kraya A.A., Piao S., Xu X., Zhang G., Herlyn M., Gimotty P., Levine B., Amaravadi R.K., Speicher D.W. (2015). Identification of secreted proteins that reflect autophagy dynamics within tumor cells. Autophagy.

[29] Wang W.J., Wang Y., Chen H.Z., Xing Y.Z., Li F.W., Zhang Q., Zhou B., Zhang H.K., Zhang J., Bian X.L. (2014). Orphan nuclear receptor TR3 acts in autophagic cell death via mitochondrial signaling pathway. Nat. Chem. Biol..

[30] Al-Qatati A., Aliwaini S. (2017). Combined pitavastatin and dacarbazine treatment activates apoptosis and autophagy resulting in synergistic cytotoxicity in melanoma cells. Oncol. Lett..

[31] Poillet-Perez L., Despouy G., Delage-Mourroux R., Boyer-Guittaut M. (2015). Interplay between ROS and autophagy in cancer cells, from tumor initiation to cancer therapy. Redox. Biol..

[32] Li Z.Y., Yang Y., Ming M., Liu B. (2011). Mitochondrial ROS generation for regulation of autophagic pathways in cancer. Biochem. Biophys. Res. Commun..

[33] Halliwell B. (2003). Oxidative stress in cell culture: An under-appreciated problem?. FEBS Lett..

[34] Babich H., Ackerman N.J., Burekhovich F., Zuckerbraun H.L., Schuck A.G. (2009). Gingko biloba leaf extract induces oxidative stress in carcinoma HSC-2 cells. Toxicol. In Vitro.

[35] Kelts J.L., Cali J.J., Duellman S.J., Shultz J. (2015). Altered cytotoxicity of ROS-inducing compounds by sodium pyruvate in cell culture medium depends on the location of ROS generation. SpringerPlus.

[36] Thor H., Smith M.T., Hartzell P., Bellomo G., Jewell S.A., Orrenius S. (1982). The metabolism of menadione (2-methyl-1,4-naphthoquione) by isolated hepatocytes. A study of the implications of oxidative stress in intact cells. J. Biol. Chem..

[37] Kwak S.Y., Jeong Y.K., Kim B.Y., Lee J.Y., Ahn H.J., Jeong J.H., Kim M.S., Kim J., Han Y.H. (2014). Dimethylacrylshikonin sensitizes human colon cancer cells to ionizing radiation through the upregulation of reactive oxygen species. Oncol. Lett..

[38] Huang J., Lam G.Y., Brumell J.H. (2011). Autophagy signaling through reactive oxygen species. Antiox. Redox. Signal.

[39] Wiench B., Eichhorn T., Paulsen M., Efferth T. (2012). Shikonin directly targets mitochondria and causes mitochondrial dysfunction in cancer cells. Evid.-Based Complement. Altern. Med..

[40] Gara R.K., Srivastava V.K., Duggal S., Bagga J.K., Bhatt M.L.B., Sanyal S., Mishra D.P. (2015). Shikonin selectively induces apoptosis in human prostate cancer cells through the endoplasmic reticulum stress and mitochondrial apoptotic pathway. J. Biomed. Sci..

[41] Han X., Kang K.A., Piao M.J., Zhen A.X., Hyun Y.J., Kim H.M., Ryu Y.S., Hyun J.W. (2018). Shikonin Exerts Cytotoxic Effects in Human Colon Cancers by Inducing Apoptotic Cell Death via the Endoplasmic Reticulum and Mitochondria-Mediated Pathways. Biomol. Ther..

[42] Liang W., Cai A., Chen G., Xi H., Wu X., Cui J., Zhang K., Zhao X., Yu J., Wei B. (2016). Shikonin induces mitochondria-mediated apoptosis and enhances chemotherapeutic sensitivity of gastric cancer through reactive oxygen species. Sci. Rep..

[43] Chang H.Y., Chi J.T., Dudoit S., Bondre D., Van de Rijn M., Botstein D., Brown P.O. (2002). Diversity, topographic differentiation, and positional memory in human fibroblasts. Proc. Natl. Acad. Sci. USA.

[44] Zhou W.J., Zhang X., Cheng C., Wang F., Wang X.K., Liang Y.J., To K.K.W., Zhou W., Huang H.B., Fu L.W. (2012). Crizotinib (PF-02341066) reverses multidrug resistance in cancer cells by inhibiting the function of P.-glycoprotein. Br. J. Pharmacol..

[45] Akiyode O., George D., Getti G., Boateng J. (2016). Systematic comparison of the functional physico-chemical characteristics and biocidal activity of microbial derived biosurfactants on blood-derived and breast cancer cells. J. Colloid Interface Sci..

[46] Peer D., Karp J.M., Hong S., Farokhzad O.C., Margalit R., Langer R. (2007). Nanocarriers as an emerging platform for cancer therapy. Nat. Nanotechnol..

[47] Li H., Tong Y., Bai L., Ye L., Zhong L., Duan X., Zhu Y. (2018). Lactoferrin functionalized PEG-PLGA nanoparticles of shikonin for brain targeting therapy of glioma. Int. J. Biol. Macromol..

[48] Kaur A., Jyoti K., Baldi A., Jain U.K., Chandra R. (2018). Self-assembled nanomicelles of amphiphilic clotrimazole glycyl-glycine analogue augmented drug delivery, apoptosis and restrained melanoma tumour progression. Mater. Sci. Eng. C Mater. Biol. Appl..

[49] Karbiener M., Pisani D.F., Frontini A., Oberreiter L.M., Lang E., Vegiopoulos A., Mössenböck K., Bernhardt G.A., Mayr T., Hildner F. (2014). MicroRNA-26 family is required for human adipogenesis and drives characteristics of brown adipocytes. Stem Cells.

[50] Pieler R., Sanchez-Cabo F., Hackl H., Thallinger G.G., Trajanoski Z. (2004). ArrayNorm: Comprehensive normalization and analysis of microarray data. Bioinformatics.

[51] Sturn A., Quackenbush J., Trajanoski Z. (2002). Genesis: Cluster analysis of microarray data. Bioinformatics.

[52] Gene Ontology Consortium (2015). Gene Ontology Consortium: Going forward. Nucleic Acids Res..

